# Intermediate phases during solid to liquid transitions in long-chain *n*-alkanes

**DOI:** 10.1039/c7cp01468f

**Published:** 2017-05-11

**Authors:** Stella Corsetti, Thomas Rabl, David McGloin, Johannes Kiefer

**Affiliations:** a SUPA, School of Science & Engineering , University of Dundee , Nethergate , Dundee , DD1 4HN , Scotland , UK . Email: s.corsetti@dundee.ac.uk; b Drug Discovery Unit , College of Life Sciences , University of Dundee , Dow Street , Dundee , DD1 5EH , Scotland , UK; c Technische Thermodynamik , Universität Bremen , Badgasteiner Str. 1 , 28359 Bremen , Germany; d School of Engineering , University of Aberdeen , Aberdeen , AB24 3UE , Scotland , UK

## Abstract

The solid to liquid phase transition of *n*-alkanes with more than ten carbon atoms is an interesting phenomenon relevant to many fields, from cosmetics to automotive. Here we report Raman spectroscopy of tetradecane, pentadecane and hexadecane as a function of temperature. In order to gain information on the structural changes that the hydrocarbons undergo during melting, and to determine the temperature and the speed at which the phase change occurs, their temperature-dependent Raman spectra are acquired. The spectra are analysed not only with respect to frequency shifts, band widths, and intensity ratio of certain bands, but also using a principal component analysis. The spectroscopic data suggest that the solid to liquid phase transition in hexadecane, differently from tetradecane and pentadecane, is almost instantaneous. Tetradecane shows a slightly faster transition than pentadecane. In addition, a rotator phase as an intermediate state between the liquid and crystalline solid phases is identified in pentadecane. Different characteristic features in the solid spectra of the hydrocarbons relate tetradecane and hexadecane to a tryclinic crystalline structure, and pentadecane to an orthorhombic structure.

## Abstract 2

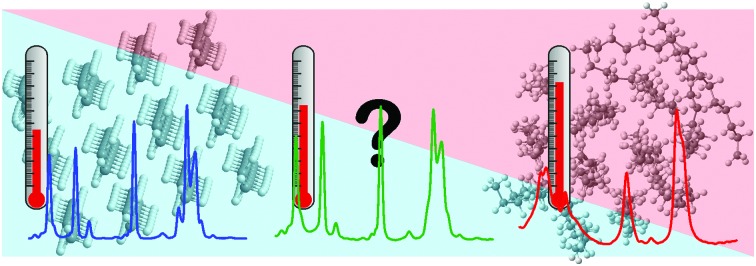
Temperature-dependent Raman spectra of tetradecane, pentadecane and hexadecane are collected and analysed to unveil the difference in the speed of their phase transitions while gaining information about their structural changes.

## 1 Introduction

The crystallisation of *n*-alkanes is an interesting subject area to explore because of its relevance to important industrial applications, such as the processing of fats, surfactants, and oils.^1^*n*-Alkanes not only represent the main building blocks of complex organic compounds, such as lipids, surfactants, liquid crystals and polymers, but are also the main constituents of traditional hydrocarbon fuels. The liquid to solid phase transitions in *n*-alkanes become particularly important for those compounds with more than ten carbon atoms as their melting point is above –30 °C. Consequently, such a transition may occur during winter time when the ambient temperature reaches the melting point leading to significant changes in the macroscopic properties of the fluid in the engine of a car.

In going from the crystalline ordered solid phase to the liquid phase, *n*-alkanes can show intermediate phases, called rotator phases. A rotator phase is a high-temperature solid phase, which exhibits long-range order in the molecular axis orientation, but lacks long range order in the rotational degree of freedom of molecules about their long axis.^2^ Hence, the molecules are free to rotate. Rotator phases have been studied during the last two decades.^3^–^9^ In general, odd-numbered *n*-alkanes with 9 < *n* < 39 and even-numbered alkanes with 20 < *n* < 38 show at least one rotator phase.^2^ A few degrees Celcius below the temperature corresponding to the rotator phase, a solid–solid phase transition occurs where the crystal phase is formed. For *n* < 30, even *n*-alkanes undergo a liquid to solid phase transition that results in the formation of a triclinic phase, while odd *n*-alkanes form an orthorhombic crystal structure.^10^ This behaviour was noted to decrease with increasing alkane chain length. A triclinic crystal system has three unequal axes all intersecting at oblique angles. An orthorhombic crystal system has three mutually perpendicular axes, coinciding with the crystallographic axes, all of different lengths. A greater stability is associated with even *n*-alkanes, which crystallize into the triclinic crystal structure. This is consistent with a heterogeneous freezing mechanism, in which the nucleation starts at the free surface of the sample, leading to surface freezing.^1^ X-ray and surface tension measurements have been used by Wu *et al.*^11^ to observe the formation of a crystalline monolayer on the surface of liquid *n*-alkanes a few degrees above the bulk solidification temperature. Later, Ocko *et al.*^12^ studied the structure of the monolayer in detail determining its existence in alkanes with carbon number (*n*) of 16 < *n* < 50.

Besides X-ray diffraction, Raman spectroscopy can be used to determine alkyl chain interactions and conformational order. In the Raman spectra of alkanes, for example, the ratio between the intensities of the asymmetric and symmetric CH_2_ stretching modes is considered to be an indicator of the rotational and conformational order.^13^ The dependance of the ratio of band amplitudes associated with CH_2_ asymmetric and symmetric modes in Raman spectra of *n*-alkanes on the lateral order of extended chains was also demonstrated.^14^,^15^

In this paper we use Raman spectroscopy to investigate and compare the solid to liquid phase transition of three different *n*-alkanes: tetradecane, pentadecane and hexadecane. In the past, Raman spectroscopy has been mainly used to investigate the solid to liquid transition of *n*-alkanes due to pressure.^16^–^19^ A few studies used Raman spectroscopy to observe structural changes in pure *n*-alkanes due to a systematic change in temperature. Brambilla *et al.*^20^ observed short intermolecular correlations activating the formation of segments of trans-planar chains in different alkanes just above and at the melting temperature. Zerbi *et al.*^21^ observed structural changes taking place at the solid–liquid phase transition and during melting in *n*-nonadecane and selectively deuterated nonadecanes. Recently, changes in the fingerprint region of the Raman spectra of the *n*-alkane CH_21_H_44_ and of some *n*-alkanes with 21 < *n* < 60 during melting were studied.^22^,^23^

Here, for the first time, we present a systematic analysis and comparison of temperature-dependent Raman spectra of tetradecane, pentadecane and hexadecane in order to unveil the difference in the speed of their phase transitions while gaining information about their structural changes. Both the fingerprint (1000–1600 cm^–1^) and the CH stretching (2700–3100 cm^–1^) regions are considered in order to have an independent comparison of the results and determine if information can be obtained equivalently from either region. The two spectral windows are first analysed by combining the detection of frequency shifts, band widths, and intensity ratio of certain bands. Thereafter, the same regions are analysed separately using a Principal Component Analysis (PCA). This chemometric method takes into account all the spectral changes in each spectral window to detect characteristic signatures that may correlated with phase changes.

## 2 Methods

### 2.1 Hydrocarbons

Tetradecane, pentadecane, and hexadecane were purchased from Sigma-Aldrich and had a purity >99%. They were used as received. The chemico-physical characteristics of these components are summarised in Table 1. Samples were prepared by placing 20 μL of the hydrocarbon under investigation between two microscope glass coverslips (0.16 mm thickness) forming a thin liquid film of ∼150 μm in height.

**Table 1:** Thermo-physical properties of tetradecane, pentadecane and hexadecane^24^

Properties	Molecular weight (kg mol^–1^)	Density liquid (kg m^–3^)	Melting point (°C)	Boiling point (°C)
Tetradecane (CH_14_H_30_)	0.198	762	5.5	252–254
Pentadecane (CH_15_H_32_)	0.212	769	8–10	270
Hexadecane (CH_16_H_34_)	0.226	773	18	287

### 2.2 Raman spectroscopy

**Fig. 1:**
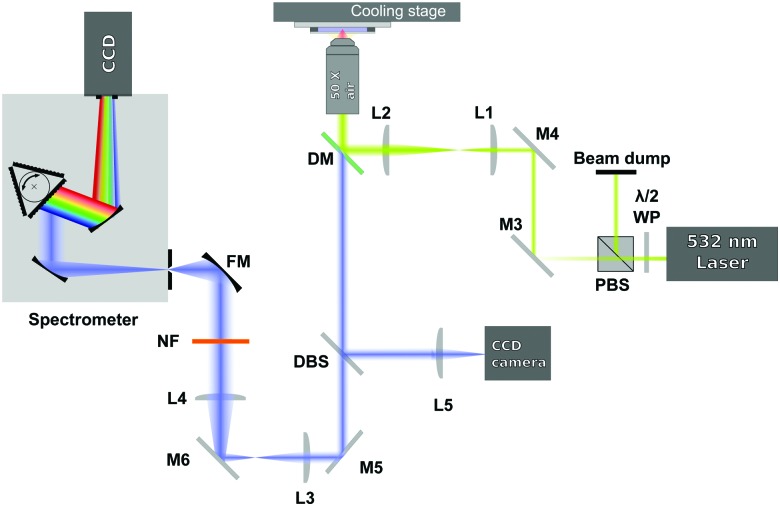
Schematic of the Raman setup used.

The spectra were acquired using a 180-degree Raman set up, as shown in Fig. 1. A 532 nm laser (Laser Quantum, Ventus Solo) was used as excitation source and it was focused on the sample (with power of ∼30 mW in the sample plane) through a 50× air microscope objective (Mitutoyo G Plan, NA = 0.5). In order to slightly overfill the back aperture of the microscope objective, the laser beam was expanded by using a 3.5 : 1 expansion telescope (L1 and L2). The laser irradiated the sample only during the acquisition of the spectra to avoid any possible laser induced heating.

The light scattered from the sample was collected by the same microscope objective used to deliver the excitation beam and then sent to a dichroic mirror (DM). The dichroic mirror acts as reflector at the laser wavelength, but transmits the Raman signal of the sample. The Raman signal then passes through a 514 nm dichroic beamsplitter (DBS). The DBS reflects a very small amount of the elastic light that leaks through the dichroic mirror to a CCD camera and lets the longer Raman shifted wavelengths pass. The signal, after being collimated by 2 achromatic lenses (L3 and L4), is then further filtered by a 532 nm notch filter (NF) and focused by a focusing mirror (FM) onto the entrance slit of a spectrograph (Andor Shamrock, entrance slit 220 μm, focal length 500 mm, grating 1200 lines per mm). The dispersed signal is detected using a CCD camera (Andor Newton DU920P-BEX2-DD). In order to optimise the Raman signal level the exposure time was varied automatically between 1 to 3.6 seconds.

Each sample was initially frozen by placing it in contact with the metallic surface of a cooling stage (PE120 Peltier System, Linkam Scientific) set at a temperature lower than the expected sample melting temperature (see Table 1). The samples were then heated at 0.1 °C min^–1^ until they became liquid. At each temperature step a pausing time of 2 minutes was set allowing the temperature in the sample to equilibrate at the temperature set in the cooling stage before acquiring the Raman spectra. The spectra, passing from the solid to the liquid state, were recorded each 0.1 °C. At the same time, the temperature of the cooling stage, which was assumed to be the same as the temperature in the thin film sample, was acquired.

Using the above system, Raman spectra of tetradecane were acquired in the temperature range between 2 and 5.8 °C. Spectra of pentadecane were acquired between 7 and 9.8 °C and between –4 and –2.2 °C, while spectra of hexadecane were recorded between 15 and 18.4 °C.

## 3 Results and discussion

In this section, two different ways of analysing the temperature dependent Raman spectra of tetradecane, pentadecane and hexadecane are used. Firstly, the Raman spectra in the CH stretching and fingerprint region are studied with respect to bands position, frequency shifts, bands widths and intensity ratios of specific bands in order to identify the phase transition temperature and to investigate characteristic changes in the spectra. Secondly, a PCA, which mathematically detects overall spectral changes due to the variation in temperature, is used in the same spectral regions to distinguish between different phases.

### 3.1 Raman spectra

#### Tetradecane

**Fig. 2:**
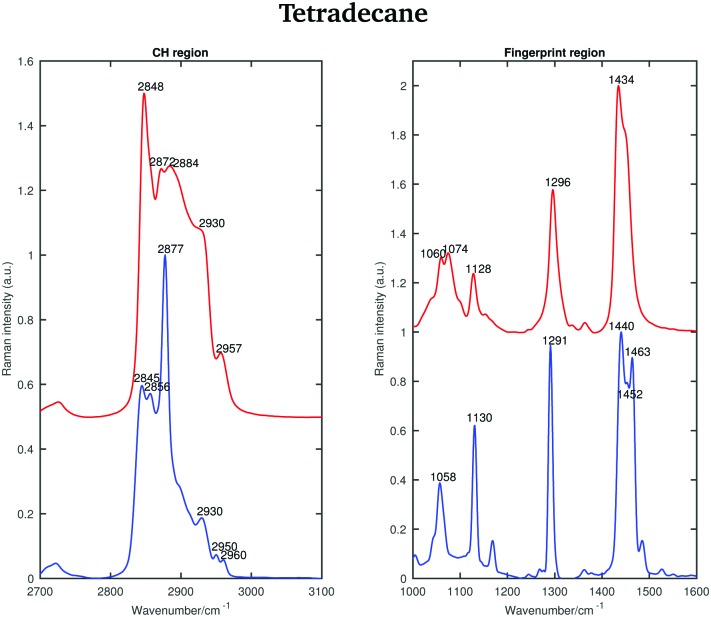
Raman spectra of solid (blue) and liquid (red) tetradecane in the CH stretching and fingerprint region acquired at 2 and 5.8 °C, respectively.

The Raman spectra of liquid and solid tetradecane (taken at 2 and 5.8 °C, respectively) in the CH stretching and in the fingerprint region are displayed in Fig. 2.

In the liquid spectrum the wavenumbers around 2848 cm^–1^ and around 2872 cm^–1^ are attributed to the symmetric and asymmetric stretching vibrations of the CH_2_ groups, respectively. The wavenumbers around 2930 cm^–1^ and around 2957 cm^–1^ are due to the symmetric and asymmetric CH_3_ stretching vibrations, respectively. There appear to be two shoulder bands in the liquid spectrum: one at the high frequency wing of the 2848 cm^–1^ band and one between the 2884 cm^–1^ and 2930 cm^–1^ bands. The Raman intensities of all CH_3_ asymmetric stretches are very small compared to the other modes and the spectral bands of these modes overlap with more intensive CH_2_ asymmetric modes. The band associated with the asymmetric CH_2_ modes at 2872 cm^–1^, which merges with the broad Fermi resonance band at 2884 cm^–1^, becomes strong and sharp and shifts to 2877 cm^–1^ in the spectrum of the solid phase. The band associated with the symmetric CH_3_ modes, mixed with a second manifold of overtones and combinations of the CH bending modes at 2930 cm^–1^, is quite broad and relatively strong in the liquid state and becomes narrower and weaker in the solid state. The band associated with the asymmetric methyl stretch at 2957 cm^–1^ in the liquid state is split into two bands at 2950 and 2960 cm^–1^ in the solid state. This split is typical for a triclinic structure.^15^ The band intensity of the symmetric CH_2_ stretching modes at 2848 cm^–1^ in the liquid spectrum decreases significantly when the sample becomes solid and red shifts to 2845 cm^–1^. On the other hand, the weak shoulder on the high frequency side of the symmetric CH_2_ modes in the spectrum of the liquid phase, becomes stronger in the solid state and it can be found at 2856 cm^–1^. The intensities of the bands at 2845 cm^–1^ and at 2856 cm^–1^ are comparable, which is another feature of a triclinic packing of methylene chains. In the hexagonal alkanes, the band at 2856 cm^–1^ becomes a weak shoulder. In monoclinic and orthorhombic alkanes, this band contributes to the broadening of the band associated with the symmetric CH_2_ modes.^15^

Considering the Raman spectra in the fingerprint region, in the liquid state the bands are broader than in the solid state, indicating a reduction in intermolecular interactions, leading to a decreased influence in vibrational motion. In the liquid state, a weak shoulder can be found at the higher frequency of the CH_2_ bending band (1434 cm^–1^). In the solid phase, the CH_2_ bending band shifts to 1440 cm^–1^ and the CH_3_ bending band becomes a well defined band at 1463 cm^–1^.

**Fig. 3:**
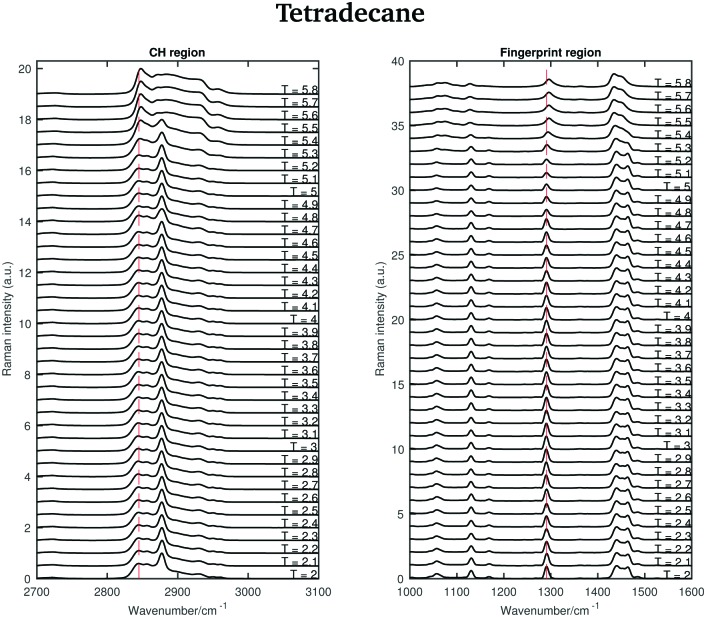
Raman spectra of tetradecane in the CH stretching and fingerprint region recorded between 2 and 5.8 °C.

The Raman spectra of tetradecane in the CH stretching and fingerprint region acquired every 0.1 °C in the temperature range between 2 and 5.8 °C are shown in Fig. 3. By a visual inspection of both regions tetradecane reach the liquid phase at 5.5 °C. This is in perfect agreement with the melting point reported in Table 1.

**Fig. 4:**
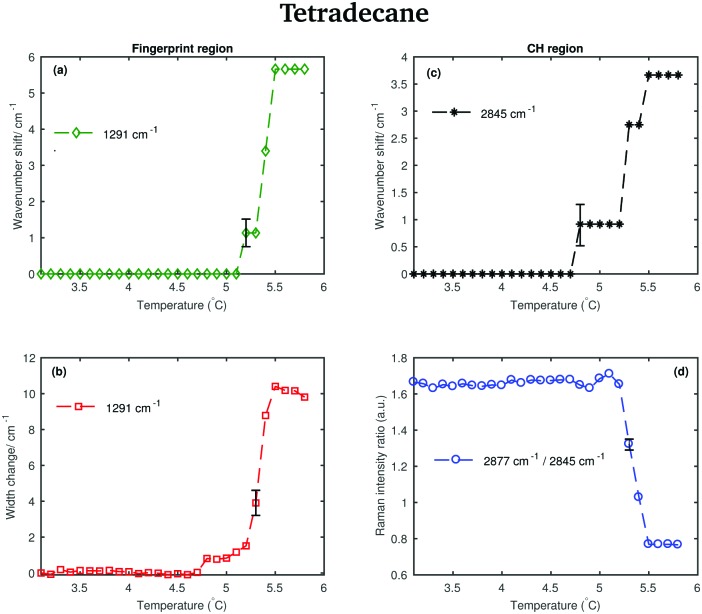
Changes in the fingerprint and CH stretching region of the Raman spectra of tetradecane as function of temperature. (a) Wavenumber shift of the twisting band (at 1291 cm^–1^ in the spectrum of the solid phase) as function of temperature. (b) Width change of the twisting band as function of temperature. (c) Wavenumber shift of the CH_2_ symmetric stretching band (at 2845 cm^–1^ in the spectrum of the solid phase) as function of temperature. (d) Intensity ratio of the CH_2_ asymmetric and symmetric stretching bands (at 2877 cm^–1^ and at 2845 cm^–1^ in the spectrum of solid tetradecane, respectively).

Different spectral changes can be observed in moving from the solid to the liquid phase in both the CH stretching and the fingerprint region. Focusing on the CH stretching region, in going from the solid to the liquid phase the CH_2_ symmetric stretching band shifts to higher wavenumbers as soon as the transition starts (see red dashed line in the CH stretching region in Fig. 3). This blue-shift becomes clearer in Fig. 4(c), which shows the frequency shift of the CH_2_ symmetric stretching band (difference of band wavenumber in a spectrum at a given temperature and the band wavenumber of the corresponding band in the spectrum of solid tetradecane at 2 °C) as function of temperature. Such a blue-shift, which indicates a stiffening of the CH covalent bond, could be due to a weakening of the intermolecular interactions during the transition to the liquid phase.

Furthermore, a net change in the CH_2_ symmetric stretching band intensity in going from the solid to the liquid phase is observed in Fig. 3. Changes in the intensity of the CH_2_ symmetric stretching band (at 2848 cm^–1^ in the liquid spectrum) are due to different chain packing involving different lateral chain interactions. The absolute value of the intensity ratio between the band at 2872 cm^–1^ (at 2877 cm^–1^ in the spectrum of the solid tetradecane) and the band at 2848 cm^–1^ (at 2845 cm^–1^ in the spectrum of the solid tetradecane) would depend on the order due to lateral crystalline interactions associated with the molecular packing. A higher ratio corresponds to chains in a more crystalline state. In Fig. 4(d) this ratio is plotted as function of temperature. The ratio in the liquid phase is found to be ∼0.7 (at 5.5 °C), and in the solid phase it is found ∼1.67. Using such ratios of intensities, a quantitative measure of the lateral order in hydrocarbon systems has been proposed by Gaber and Peticolas,^14^ as given by the following expression:1
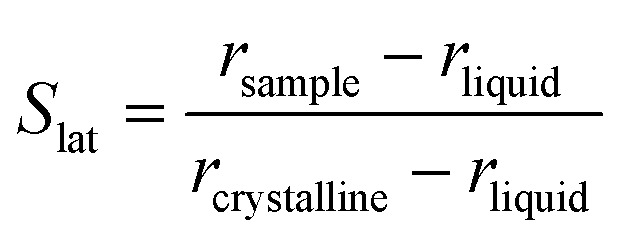
where *r*_sample_ and *r*_liquid_ are the Raman intensity ratios of the bands associated with the asymmetric and symmetric CH_2_ stretching modes in the sample in an intermediate phase between the solid and the liquid phase and in the liquid phase, respectively. *r*_crystalline_ is the value of the intensity ratio in the crystalline state. In terms of this relation, *S*_lat_ = 1 will correspond to chains in the crystalline state, whereas *S*_lat_ = 0 corresponds to chains in the liquid phase.

Considering the fingerprint region, a blue-shift of the band associated with the twisting vibrational modes (see red dashed line in the fingerprint region in Fig. 3) and a broadening of the twisting band in passing from the solid to the liquid phase is observed. Further changes are visible in the CH bending region.

The data in Fig. 4(a) show the shift of the band associated to the twisting modes (difference of band wavenumber in a spectrum at a given temperature and the band wavenumber of the corresponding band in the spectrum of solid tetradecane at 2 °C) as function of temperature.

The data in Fig. 4(b) show the change in width of the twisting band (difference between the width of the band in a spectrum at a given temperature and the width of the same band in the spectrum of solid tetradecane at 2 °C) as function of temperature. In passing from the solid to the liquid phase a broadening of the band, indicating a reduction in intermolecular interactions, is observed. In conclusion, the data in Fig. 4 confirm that tetradecane is completely liquid at 5.5 °C as visually observed in Fig. 3, and as suggested by the data in Table 1. However, spectral changes in the CH stretching and the fingerprint region can be detected earlier between 4.8 and 5.1 °C. This shows that the transition from the solid to the liquid phase is not instantaneous, but it happens through a series of uncharacterised intermediate phases.

#### Pentadecane

**Fig. 5:**
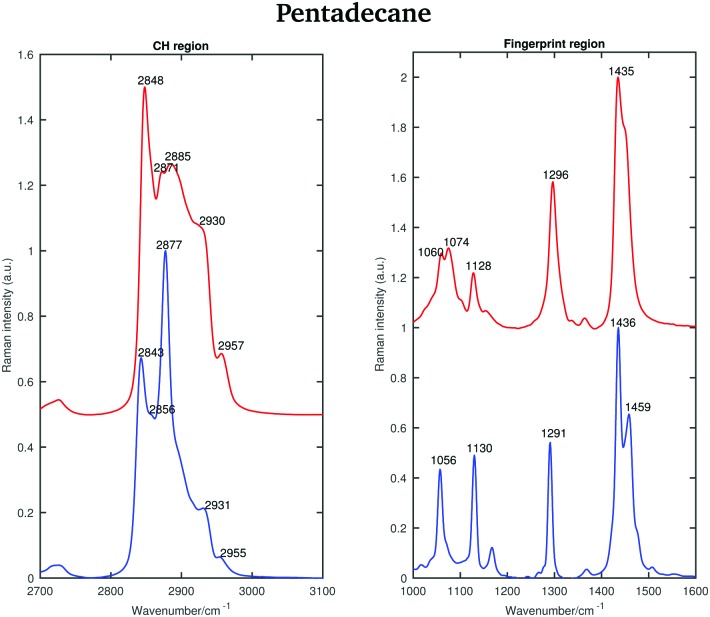
Raman spectra of solid (blue) and liquid (red) pentadecane in the CH stretching and the fingerprint region recorded at 7 °C and 9.8 °C, respectively.

Fig. 5 shows the Raman spectra of liquid and solid pentadecane (taken at 7 °C and 9.8 °C) in the CH stretching and fingerprint region. The symmetric and asymmetric CH_2_ modes in the liquid pentadecane spectrum are observed at 2848 and at 2871 cm^–1^, respectively. The CH_3_ symmetric and asymmetric modes are observed at 2930 and at 2957 cm^–1^, respectively. The band at 2885 cm^–1^ is due to Fermi resonance, similar to the spectrum of tetradecane. In the solid spectrum the band associated with the CH_2_ symmetric modes decreases in intensity and can be found at 2843 cm^–1^. The band associated with the asymmetric CH_2_ stretching modes becomes narrower and shifts to 2877 cm^–1^. Both the symmetric and asymmetric CH_3_ modes become weaker and shift to 2931 and 2955 cm^–1^, respectively. As for tetradecane, the ratio between the asymmetric and the symmetric CH_2_ stretching modes increases when passing from the liquid to the solid phase. However, contrary to tetradecane, the intensity of the symmetric CH_2_ stretching modes, and the weak shoulder observed at 2856 cm^–1^ in the spectrum of solid pentadecane are not comparable. Furthermore, no splitting of the CH_3_ asymmetric modes is observed in the solid phase. This indicates that pentadecane does not solidify in a triclinic phase. It is in fact known that it solidifies in an orthorhombic phase.^25^ Typical of this phase is the conservation of the shape of the symmetric CH_2_ modes in the liquid and solid spectra.^26^

As for tetradecane, the bands in the fingerprint region of the liquid spectrum are broader than in the solid one. In the liquid spectrum, the CH_2_ bending band and the Fermi resonance band can be found at 1435 cm^–1^ and 1445 cm^–1^, respectively.

**Fig. 6:**
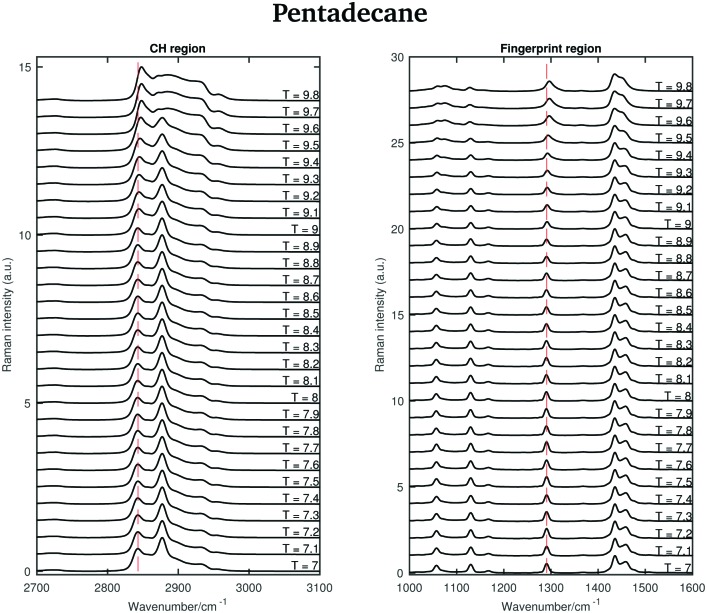
Raman spectra of pentadecane in the CH stretching and fingerprint region recorded between 7 and 9.8 °C.

Fig. 6 shows the Raman spectra of pentadecane in the CH stretching and in the fingerprint region acquired every 0.1 °C between 7 and 9.8 °C. As in the tetradecane spectra, in the CH stretching region a blue-shift of the CH_2_ symmetric stretching band is observed when passing from the solid to the liquid phase. Also a blue-shift and a broadening of the twisting band in the fingerprint region is visible. A visual inspection of both regions indicates that pentadecane reaches the liquid phase at 9.6 °C.

**Fig. 7:**
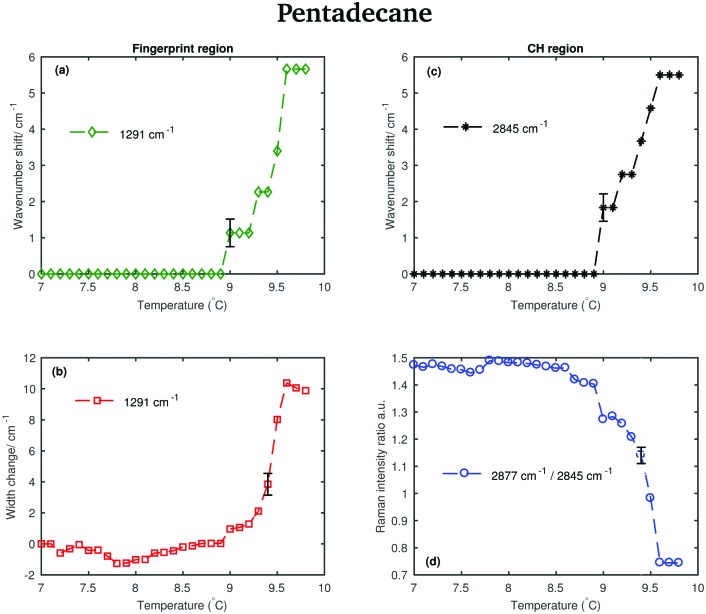
Changes in the fingerprint and CH stretching region of the Raman spectra of pentadecane as function of temperature. (a) Wavenumber shift of the twisting band (at 1291 cm^–1^ in the spectrum of solid pentadecane) as function of temperature. (b) Width change of the twisting band as function of temperature. (c) Wavenumber shift of the CH_2_ symmetric stretching band (at 2843 cm^–1^ in the spectrum of the solid phase) as function of temperature. (d) Intensity ratio of the CH_2_ asymmetric and symmetric stretching bands (at 2877 cm^–1^ and at 2843 cm^–1^ in the spectrum of solid pentadecane, respectively).

The shifting of the band associated with the twisting vibrational modes, the change in width of the twisting band, the shifting of the symmetric CH_2_ band and the intensity ratio of the asymmetric and symmetric CH_2_ bands in passing from the solid to the liquid phase are displayed in Fig. 7.

The data in Fig. 7(a) and (b) show that spectral changes can be detected in both the CH stretching and the fingerprint region of pentadecane from 8.9 °C and that pentadecane reaches the liquid phase at 9.6 °C as confirmed by the data in Fig. 7(d). This is in agreement with the melting temperature range given in Table 1. The length of the transition phase in pentadecane is comparable to the one of tetradecane.

**Fig. 8:**
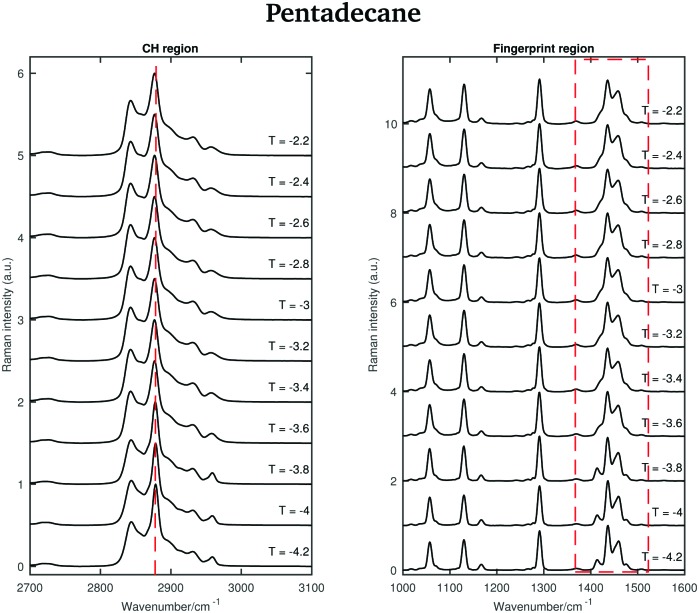
Raman spectra of pentadecane in the temperature range between –4.2 and –2.2 °C.

An odd-numbered *n*-alkane with *n* = 15, pentadecane is expected to show at least one rotator phase in turning from the crystalline ordered solid phase to the liquid phase.^2^ The presence of this solid to solid phase transition can be observed in Fig. 8 in which the Raman spectra of pentadecane taken every 0.2 °C between –4.2 °C and –2.2 °C are shown. While changes in the CH region spectra as function of temperature are subtle, in the fingerprint region the CH bending band shows a more prominent change in shape at –3.6 °C, suggesting a transition from the crystalline solid phase to a rotator phase. Pentadecane can be found in this rotator phase until it starts turning to the liquid phase (see Fig. 6). The presence of a solid to solid phase transition is also suggested by a sudden frequency shift of the CH_2_ asymmetric stretching band towards lower wavenumbers as the temperature increases (see red dashed line in the CH region).

**Fig. 9:**
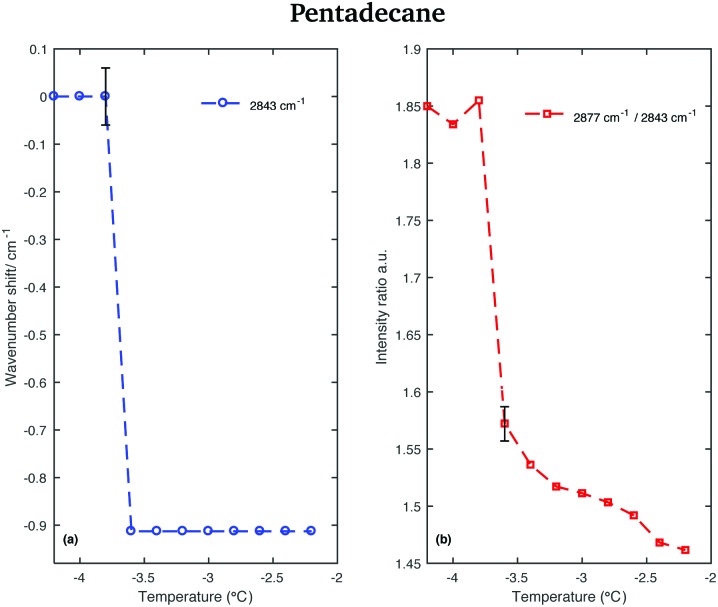
Changes in the CH stretching region of the Raman spectra of pentadecane in the temperature range between –4.2 and –2.2 °C. (a) Wavenumber shift of the CH_2_ asymmetric stretching band (at 2877 cm^–1^ at –4.2 °C) as function of temperature. (b) Intensity ratio of the CH_2_ asymmetric and symmetric stretching bands (at 2877 cm^–1^ and at 2843 cm^–1^ at –4.2 °C, respectively).

This red-shift is highlighted in Fig. 9(a), which shows the wavenumber shift of the band associated with the CH_2_ asymmetric stretching modes (difference of band wavenumber in a spectrum at a given temperature and the band wavenumber of the corresponding band in the spectrum taken at –4.2 °C) as function of temperature. A net shift can be observed at –3.6 °C. A change in the crystalline phase of the sample at –3.6 °C is also confirmed from the data in Fig. 9(b). It shows the intensity ratio of the asymmetric CH_2_ and the symmetric CH_2_ stretching bands as function of temperature.

#### Hexadecane

**Fig. 10:**
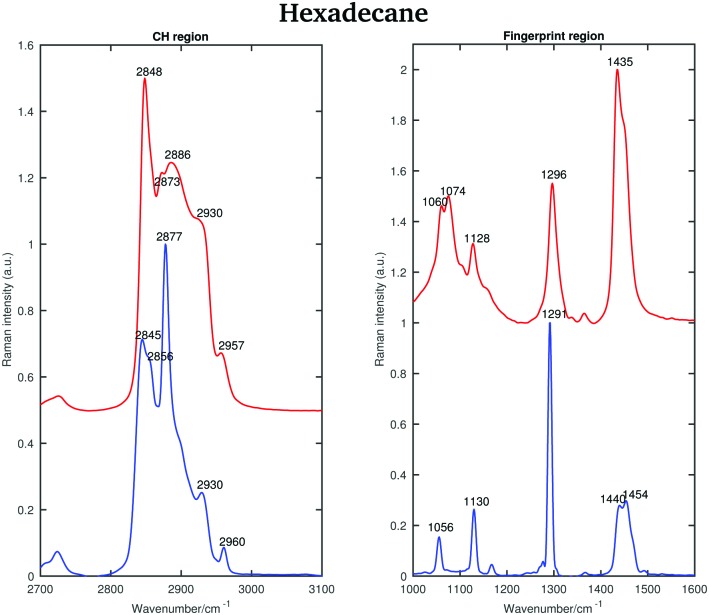
Raman spectra of solid (blue) and liquid (red) hexadecane in the CH stretching and fingerprint regions acquired at 15 and 18.4 °C, respectively.

Fig. 10 shows the Raman spectra of solid and liquid hexadecane in the CH stretching and fingerprint vibrational regions at 15 and 18.4 °C, respectively.

**Fig. 11:**
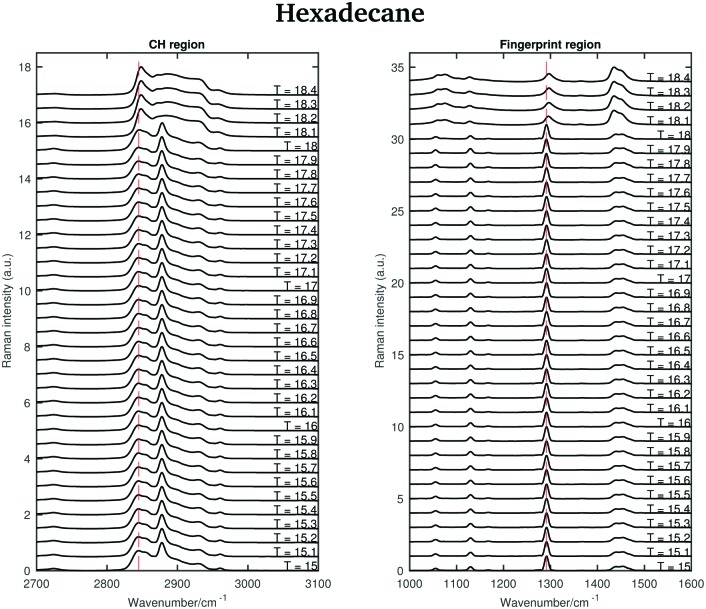
Raman spectra of solid and liquid hexadecane in the CH stretching and fingerprint region acquired in the temperature range between 15 and 18.4 °C.

The asymmetric CH_2_ band at 2873 cm^–1^, which merges with the broad Fermi resonance band at 2886 cm^–1^ in the liquid spectrum becomes strong and sharp and shifts to 2877 cm^–1^ in the solid spectrum. The band associated with the symmetric CH_3_ modes, mixed with Fermi resonance, at 2930 cm^–1^, which is quite broad and relatively strong in the liquid state, becomes narrower and weaker in the solid state. The band corresponding to the asymmetric CH_3_ modes at 2957 cm^–1^, in the liquid state, increases in intensity and becomes narrower in the solid state. Moreover, it shifts to 2960 cm^–1^. The band intensity of the symmetric CH_2_ stretching modes at 2848 cm^–1^, in the liquid spectrum, decreases significantly when the sample becomes solid and it shifts to 2845 cm^–1^. On the other hand, a weak shoulder that can be observed on the high frequency side of the symmetric CH_2_ modes in the liquid state, becomes stronger in the solid state and it can be found at 2856 cm^–1^. The band at 2856 cm^–1^ has a slightly lower intensity with respect to the symmetric CH_2_ modes. The packing in the solid hexadecane is known to be triclinic and this is confirmed by the comparable intensity of the bands at 2845 cm^–1^ and at 2856 cm^–1^, as in tetradecane. However, the splitting of the asymmetric CH_3_ modes observed in tetradecane is not present. In the liquid spectrum of hexadecane in the fingerprint region the Fermi resonance band at 1448 cm^–1^ is broad and appears only as a shoulder on the high frequency side of the CH_2_ bending band at 1435 cm^–1^. In the solid spectrum it becomes a well-defined band at 1454 cm^–1^ and has comparable intensity with the CH_2_ band. In the solid state, the CH bending band decreases in intensity. As in the other two *n*-alkanes the bands in the solid spectrum are narrower than in the liquid one. Fig. 11 shows the spectra of hexadecane in the CH stretching and fingerprint region in the temperature range between 15 and 18.4 °C. As for tetradecane and pentadecane, a blue-shift of the symmetric CH_2_ band in the CH stretching region, and of the twisting band in the fingerprint region is observed when passing from the solid to the liquid phase. Visually, the transition to the liquid phase seems to happen quite suddenly at 18.1 °C.

**Fig. 12:**
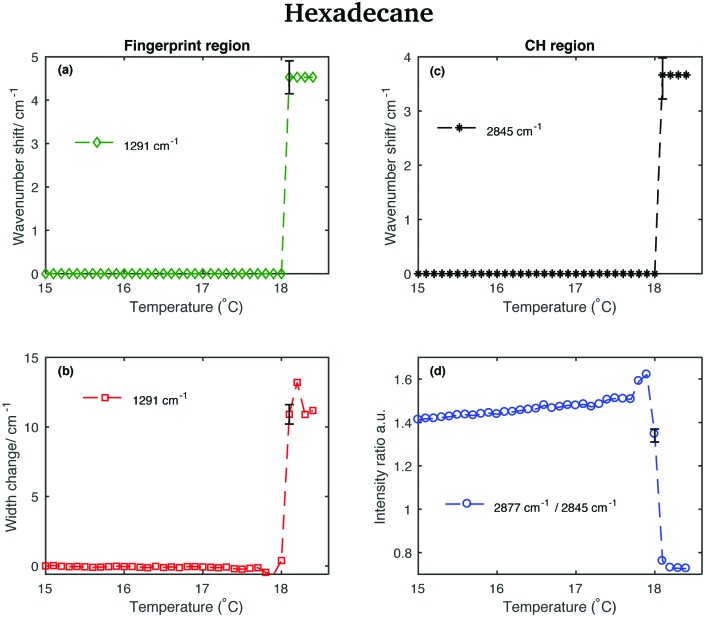
Changes in the fingerprint and CH stretching region of the Raman spectra of hexadecane as function of temperature. (a) Wavenumber shift of the twisting band (at 1291 cm^–1^ in the spectrum of solid hexadecane) as function of temperature. (b) Width change of the twisting band as function of temperature. (c) Wavenumber shift of the CH_2_ symmetric stretching band (at 2845 cm^–1^ in the spectrum of solid hexadecane) as function of temperature. (d) Intensity ratio of the CH_2_ asymmetric and symmetric stretching bands (at 2877 cm^–1^ and at 2845 cm^–1^ in the spectrum of solid hexadecane, respectively).

This is confirmed by the data in Fig. 12 that show the shifting of the band associated to the twisting vibrational modes (Fig. 12(a)), the change in width of the twisting band (Fig. 12(b)), the shifting of the symmetric CH_2_ band (Fig. 12(c)), and the change in the intensity ratio of the asymmetric and symmetric CH_2_ bands (Fig. 12(d)) during the transition from the solid to the liquid phase. In hexadecane, in contrast to tetradecane and pentadecane, the transition from the solid to the liquid phase seems to be almost instantaneous without intermediate phases.

### 3.2 Principal component analysis (PCA)

The Raman spectra of tetradecane, pentadecane, and hexadecane acquired at different temperatures were further analysed using PCA. This multivariate method, which has already been described in a previous paper,^27^ identifies spectral characteristics that describe the variance of the data set. In other words, the principal components contributing to the individual spectra are extracted. PCA was performed separately on the CH stretching and fingerprint region data sets to have an independent comparison of results obtained from each region. Before running the PCA, each spectrum was processed to remove cosmic rays and normalised to the intensity of the maximum band in the spectrum. Matlab was used to perform all data processing.

#### Tetradecane

**Fig. 13:**
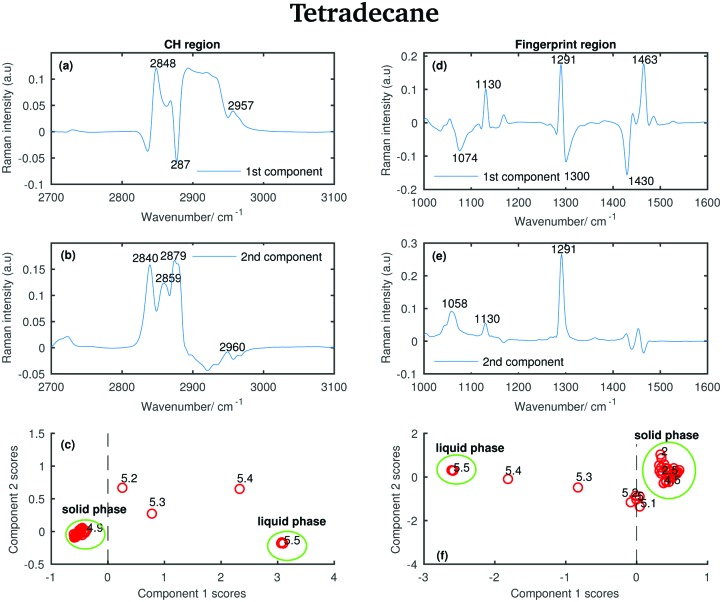
PCA of the tetradecane spectra recorded between 2 and 5.8 °C and shown in Fig. 2. (a) First PCA component for the CH stretching region. (b) Second PCA component for the CH stretching region. (c) PCA scores associated to the PCA components for the CH stretching region. (d) First PCA component for the fingerprint region. (e) Second PCA component for the fingerprint region. (f) PCA scores associated to the PCA components for the fingerprint region.

Firstly, the PCA was used to analyse the Raman spectra of tetradecane recorded between 2 and 5.8 °C (see Fig. 3). For the spectra collected in both the CH stretching and the fingerprint region, the most significant source of spectral variability in each data set is represented by the first two PCA components, which are plotted in Fig. 13. In the CH region the first two components explain 97.8% and 1.73% of variability, respectively. In the fingerprint region they explain 68.9% and 23.2% of variability, respectively.

Comparing the data in Fig. 2 with the bands in the PCA components shows that those bands arise from the variability in the Raman intensity of the bands in the original data set; therefore, a molecular origin to the features in the components can be assigned. The PCA components contain positive and negative features. An higher amount of the positive features and a lower amount of the negative ones is present in the spectra to which higher PCA scores are assigned.

The positive features in the first PCA component for the CH stretching region (Fig. 13(a)) correspond to the symmetric CH_2_ stretching vibrations at 2848 cm^–1^ and to the asymmetric CH_3_ stretching vibrations at 2957 cm^–1^ in the spectrum of the liquid phase. The negative band corresponds to the asymmetric CH_2_ modes in the spectrum of the solid phase.

The positive features in the first PCA component for the fingerprint region (Fig. 13(d)) correspond to the C–C stretching at 1130 cm^–1^, the twisting modes at 1291 cm^–1^ and the bending modes at 1463 cm^–1^ in the spectrum of the solid phase, while the negative features correspond to the bands in the spectrum of the liquid phase.

The positive and negative features in the second PCA component for the CH stretching region (Fig. 13(b)) are more difficult to assign and they most likely arise from the convolution of the bands in the spectra of the liquid and the solid phase during the transition phase.

The positive features in the second PCA component for the fingerprint region (Fig. 13(e)) correspond, as in the first component, to the bands in the spectrum of the solid phase.

The PCA scores (Fig. 13(c) and (f)) indicate how much of the variability explained by the first components is present in each of the tetradecane spectra in each spectral window. Positive scores associated with the first component in the CH stretching region (Fig. 13(c)) are correlated with increased liquid phase, and negative scores with increased solid phase. Conversely, positive scores associated with the first component in the fingerprint region (Fig. 13(f)) are correlated with increased solid phase, and negative scores with increased liquid phase.

In both Fig. 13(c) and (f), the solid and liquid phase can be clearly distinguished. In both cases, tetradecane seems to reach the liquid phase at 5.5 °C, confirming what was observed in the conventional analysis of the spectra in the previous section. If the CH stretching region is considered, the transition from the solid to the liquid phase clearly starts at 5.2 °C. If the fingerprint region is considered the transition seems starting earlier, at ∼4.8 °C.

#### Pentadecane

A PCA was also used also to analyse the Raman spectra of pentadecane recorded between 7 and 9.8 °C, see Fig. 6. Like for tetradecane, the two first principal components explain most of the variability in the pentadecane spectra collected in both the CH stretching and fingerprint region. In the CH region the first two components explain 95.7% and 4.1% of variability, respectively. In the fingerprint region they explain 75.2% and 21% of variability, respectively.

**Fig. 14:**
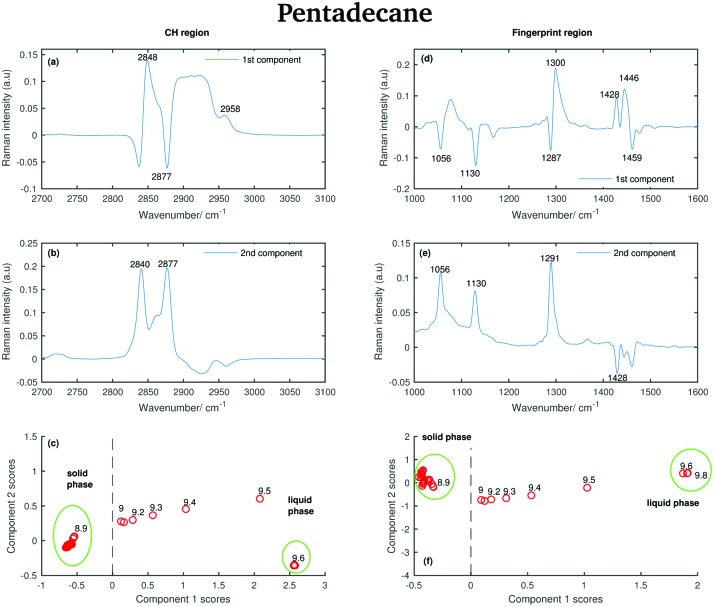
PCA of the pantadecane spectra recorded between 7 and 9.8 °C and shown in Fig. 6. (a) First PCA component for the CH stretching region. (b) Second PCA component for the CH stretching region. (c) PCA scores associated to the PCA components for the CH stretching region. (d) First PCA component for the fingerprint region. (e) Second PCA component for the fingerprint region. (f) PCA scores associated to the PCA components for the fingerprint region.

The positive features in the first pentadecane PCA component for the CH stretching region (Fig. 14(a)) retain the same meaning as the tetradecane first PCA component in the CH stretching region. In the first PCA component for the fingerprint region (Fig. 14(d)) most of the negative features can be assigned to the bands in the spectrum of the solid phase. In particular, the bands at 1056 cm^–1^, at 1130 cm^–1^ and at 1459 cm^–1^. The positive features are most likely due to the convolution of the bands in the spectra of the liquid phase and the bands in the spectra taken during the transition from the solid to the liquid phase.

The positive and negative features in the second PCA component for the CH stretching region (Fig. 14(b)) are most likely due to the convolution of the bands in the spectra of the liquid and the solid phase during the transition phase as in the case of tetradecane.

The positive features in the second PCA component for the fingerprint region (Fig. 14(e)) correspond to the bands in the spectrum of the solid phase.

The data in Fig. 14(c) show that positive scores associated to the first component in the CH stretching region are correlated with increased liquid phase, and negative scores with the solid phase. In the same way, positive scores associated to the first component in the fingerprint region (Fig. 14(f)) are correlated with increased liquid phase, and negative scores with the solid phase. In both regions, the liquid and solid phase are well distinguishable.

In contrast to tetradecane, considering either the CH stretching or the fingerprint region, the transition from the solid to the liquid phase in pentadecane starts at 9 °C, and it results to be liquid at 9.6 °C, confirming the above observations. From the PCA it is even more clear that the transition from the solid to the liquid phase takes longer in pentadecane than in tetradecane.

**Fig. 15:**
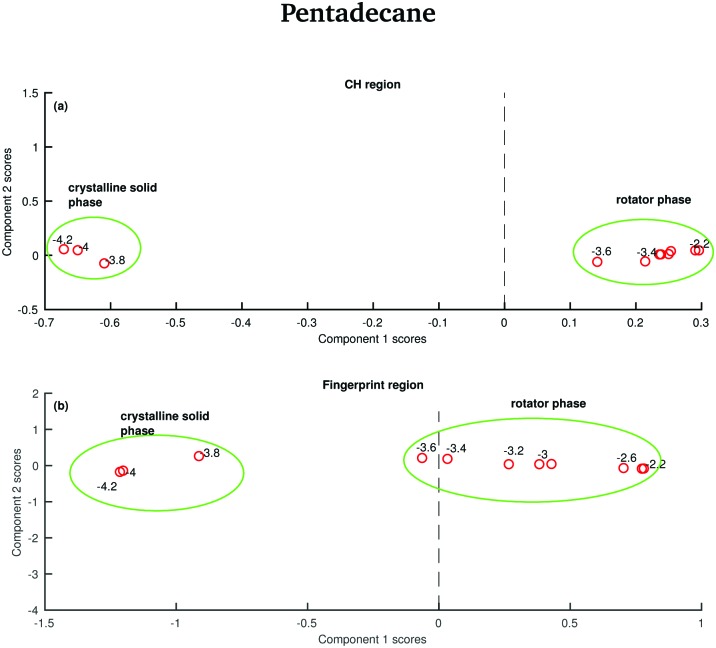
PCA of the pentadecane spectra recorded between –4.2 and –2.2 °C and shown in Fig. 8. (a) PCA scores associated to the CH stretching region. (b) PCA scores associated to the fingerprint region.

The data in Fig. 15 show the score plots derived from the PCA of the spectra of pentadecane recorded between –4.2 and –2.2 °C (see Fig. 9). The two different solid phases can be distinguished and the transition between the crystalline solid phase to the rotator phase occurs at –3.6 °C. The two phases can be better distinguished if the PCA is done on the CH stretching region even though a more dramatic change seems to be observed in the fingerprint region.

**Fig. 16:**
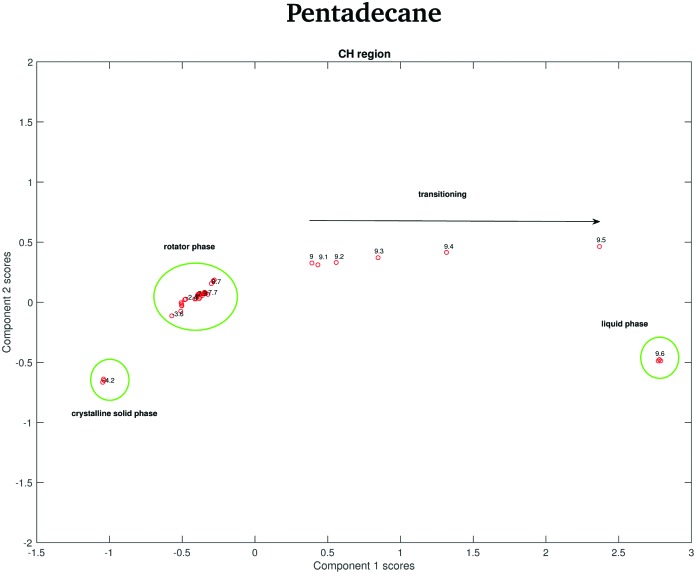
PCA scores associated to the PCA of the CH stretching region of both the pentadecane spectra recorded between –4.2 and –2.2 °C and between 7 and 9.8 °C.

In order to verify if the rotator phase coincides with the solid phase observed between 7 and 9.8 °C, a PCA considering as input data both the data recorded between –4.2 and –2.2 °C and the data recorded between 7 and 9.8 °C is carried out. In this case, only the CH stretching region, which is the one better predicting the presence of two different solid phases between –4.2 and –2.2 °C is considered. The score plot, shown in Fig. 16, suggests that the rotator phase occurring at –3.6 °C lasts until pentadecane begins to melt at 9 °C.

#### Hexadecane

**Fig. 17:**
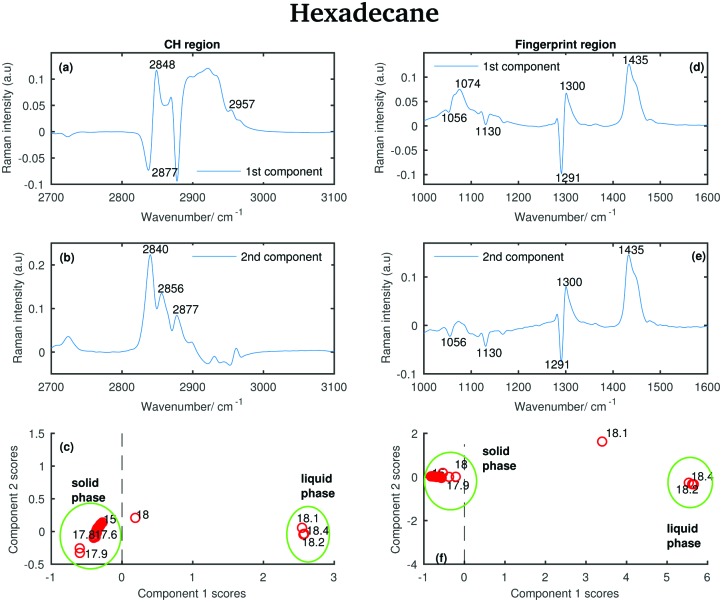
PCA of the hexadecane spectra recorded between 15 and 18.4 °C and shown in Fig. 11. (a) First PCA component for the CH stretching region. (b) Second PCA component for the CH stretching region. (c) PCA scores associated to the PCA components for the CH stretching region. (d) First PCA component for the fingerprint region. (e) Second PCA component for the fingerprint region. (f) PCA scores associated to the PCA components for the fingerprint region.

Fig. 17 shows the results from the PCA of the Raman spectra of hexadecane acquired between 15 and 18.4 °C (see Fig. 6). As for tetradecane and pentadecane, only two principal components for both the CH stretching and the fingerprint region are retained to explain the variability in the data sets. In the CH region the first two components explain 98.6% and 1.2% of variability, respectively. In the fingerprint region they explain 97.3% and 2.3% of variability, respectively.

Some of the positive features in the first hexadecane PCA component for the CH stretching region (Fig. 17(a)) can be assigned to the vibrational modes identified in the spectrum of the liquid phase (see the band at 2848 and 2957 cm^–1^ corresponding to the symmetric CH_2_ modes and to the asymmetric CH_3_ modes, respectively). The negative band at 2877 cm^–1^ is assigned to the asymmetric CH_2_ modes in the spectrum of the solid phase.

In the first PCA component for the fingerprint region (Fig. 17(d)) most of the positive features can be assigned to the bands in the spectrum of the liquid phase, while the negative ones corresponds to the bands in the spectrum of the solid phase.

The positive features in the second PCA component for the CH stretching region (Fig. 17(b)) corresponds to bands that can be found in both the spectra of the liquid and the solid phase.

The features in the second PCA component for the fingerprint region (Fig. 17(e)) retain the same meaning as the features in the first component for the same region.

The scores plots in Fig. 17(c and f) show as for pentadecane that positive scores associated to the first component in both regions are correlated with increased liquid phase, and negative scores with the solid phase. However, in contrast to the other two hydrocarbons, the transition from the solid to the liquid phase is almost instantaneous (it takes less than 0.1 °C) showing hexadecane to be totally liquid at 18.1 °C if the CH stretching region is considered and at 18.2 °C if the fingerprint region is analysed.

## 4 Conclusion

In this paper we investigated the solid to liquid phase transition of tetradecane, pentadecane, and hexadecane analysing their temperature dependent Raman spectra.

Spectra were analysed using conventional spectra analysis, *i.e.* detecting wavenumber shifts, band widths, and intensity ratio of certain bands together with Principal Component Analysis. Using conventional spectra analysis common characteristics between the Raman spectra of even and odd *n*-alkanes have been detected. The spectra of all the hydrocarbons show a blue-shift when passing from the solid to the liquid phase. This can be attributed to the fact that the bonds in the solid phase are more rigid making molecules vibrate at lower frequencies. Another common characteristic is that the ratio between the asymmetric and the symmetric CH_2_ stretching modes increases when the *n*-alkanes become liquid. In addition, specific features of even and odd *n*-alkanes have also been observed, for example, a net decrease of the band intensity of the CH_2_ stretching band when passing from the liquid to the solid phase in even *n*-alkanes. Furthermore, the weak shoulder on the high frequency side of the symmetric CH_2_ modes, in the liquid state, becomes stronger and red-shifted in the solid state. Another important feature in the spectra of even *n*-alkanes was that the band associated with the asymmetric CH_3_ stretching splits into two when passing from the liquid to the solid phase. This split is typical of a triclinic crystal structure. However, the splitting was only observed in the spectra of tetradecane, but not in the ones of hexadecane, even though it is known to have the same triclinic structure. No splitting of the band associated with the CH_3_ asymmetric modes was observed in the Raman spectra of pentadecane. Odd *n*-alkanes, in fact, do not solidify in a triclinic phase, but in an orthorhombic phase. A distinct feature of this phase is the conservation of the shape of the symmetric CH_2_ when passing from liquid to solid.

By combining conventional spectral analysis with PCA we correlated spectral changes with molecular changes caused by the variation in temperature identifying the temperatures at which the hydrocarbons start melting. While tetradecane and pentadecane take longer to transition (between ∼4.8 and 5.5 °C and between 9 and 9.6 °C, respectively), hexadecane shows an almost instantaneous change in phase at 18.1 °C.

The combination of the two different approaches for data evaluation allowed the identification of rotator phases as intermediate states between liquid and crystalline solid. A rotator phase was in fact detected by analysing the spectra of pentadecane in the temperature range between –4 and –2.2 °C.

In conclusion, Raman spectroscopy is a very valuable tool to study phase transitions in hydrocarbons and the combination of conventional spectral analysis and PCA is helpful to gain insights.

## References

[cit1] Taggart A., Voogt F., Clydesdale G., Roberts K. (1996). Langmuir.

[cit2] Yamashita M., Hirao A., Kato M. (2011). J. Chem. Phys..

[cit3] Sirota E., King Jr H., Singer D., Shao H. H. (1993). J. Chem. Phys..

[cit4] Yamamoto T., Nozaki K., Hara T. (1990). J. Chem. Phys..

[cit5] Denicolo I., Doucet J., Craievich A. (1983). J. Chem. Phys..

[cit6] Doucet J., Denicolo I., Craievich A., Germain C. (1984). J. Chem. Phys..

[cit7] Barnes J., Fanconi B. (1972). J. Chem. Phys..

[cit8] Yamamoto T. (1988). J. Chem. Phys..

[cit9] Gang H., Gang O., Shao H. H., Wu X., Patel J., Hsu C., Deutsch M., Ocko B., Sirota E. (1998). J. Phys. Chem. B.

[cit10] Small D. M., Physical chemistry of lipids, Plenum Press, 1986.

[cit11] Wu X., Sirota E., Sinha S., Ocko B., Deutsch M. (1993). Phys. Rev. Lett..

[cit12] Ocko B., Wu X., Sirota E., Sinha S., Gang O., Deutsch M. (1997). Phys. Rev. E: Stat. Phys., Plasmas, Fluids, Relat. Interdiscip. Top..

[cit13] Orendorff C. J., Ducey M. W., Pemberton J. E. (2002). J. Phys. Chem. A.

[cit14] Gaber B. P., Peticolas W. L. (1977). Biochim. Biophys. Acta, Biomembr..

[cit15] Snyder R., Hsu S., Krimm S. (1978). Spectrochim. Acta, Part A.

[cit16] Wong P., Chagwedera T., Mantsch H. (1987). J. Chem. Phys..

[cit17] Er-Wei Q., Hai-Fei Z., Bei X. (2009). Chin. Phys. Lett..

[cit18] Jian X., Zheng H. (2009). Spectrochim. Acta, Part A.

[cit19] Schoen P., Priest R., Sheridan J., Schnur J. (1979). J. Chem. Phys..

[cit20] Brambilla L., Zerbi G. (2005). Macromolecules.

[cit21] Zerbi G., Magni R., Gussoni M., Moritz K. H., Bigotto A., Dirlikov S. (1981). J. Chem. Phys..

[cit22] Jin Y., Kotula A. P., Hight Walker A. R., Migler K. B., Lee Y. J. (2016). J. Raman Spectrosc..

[cit23] Kotula A. P., Walker A. R. H., Migler K. B. (2016). Soft Matter.

[cit24] Linstrom P. J. and Mallard W. G., NIST Chemistry webbook, NIST standard reference database No. 69, 2001.

[cit25] Vélez C., de Zárate J. M. O., Khayet M. (2015). Int. J. Therm. Sci..

[cit26] Snyder R. G. (1961). J. Mol. Spectrosc..

[cit27] Corsetti S., McGloin D., Kiefer J. (2016). Fuel.

